# Epidemiology of monoclonal gammopathy in Morocco – A hospital‐based study

**DOI:** 10.1002/cnr2.1814

**Published:** 2023-03-31

**Authors:** Zohra Ouzzif, Kamal Doghmi, Nezha Messaoudi, Sanae Bouhsain, Samira El Machtani, Asmae Biaz, Achraf Rachid, Abdallah Dami, Ahmed Bezza, Aissam El Maataoui

**Affiliations:** ^1^ Faculty of Medicine and Pharmacy The Military Hospital Instruction Mohamed V/Mohamed V University Rabat Morocco; ^2^ Clinical Chemistry Department, Faculty of Medicine and Pharmacy Ibn Zohr University, Agadir Teaching Hospital Agadir Morocco

**Keywords:** epidemiology, monoclonal gammopathy, monoclonal protein, Morocco

## Abstract

**Background:**

Monoclonal gammopathies are a group of disorders associated with clonal proliferation of plasma cells that produces a monoclonal protein.

**Aims:**

The main objective of this study was to describe the epidemiological and immunochemical characteristics of monoclonal gammopathies diagnosed during 19 years in a Moroccan teaching hospital.

**Materials and Results:**

This retrospective study enrolled 443 Moroccan patients with monoclonal gammopathy, patients meeting the inclusion and exclusion criteria in at the biochemistry department of Military Hospital in Rabat, the capital of Morocco, from January 2000 to August 2019. Of the 443 enrolled patients, 320 (72.23%) were men and 123 (27.77%) were women. All patients were of Caucasian origin, from 12 Moroccan regions. The patient's samples were collected and subjected to serum protein electrophoresis and serum immunofixation electrophoresis to further characterize the monoclonal protein. The mean ± *SD* age of the 443 participants was 62.24 ± 13.14 years. Reasons for being admitted to the hospital were as follows, bone pain (41.60%), renal failure (19.08%), alteration of the general condition (12.21%), and anemia (10.69). Plasma cell proliferative disorders in our study were as follows, multiple myeloma (MM) (45.65%), Monoclonal gammopathies of undetermined significance (MGUS) (39.05%), Waldenstrom's macroglobulinemia (5.58%), Lymphoma (2.27% + 1.2%), Chronic Lymphocytic Leukemia (2.48%), Plasma cell leukemia (1.86%), Plasmacytoma (0.62%), POEMS syndrome (0.41%), and Amyloidosis (0.84%). The most frequent isotypes in MM were the IgGκ (62) 36.5%, IgGλ (52) 30.6%, IgAκ (27) 15.9%, and the IgAλ (19) 11.2%. It is also worth noting that Free light chain MM represents 20% of all cases of MM.

**Conclusions:**

We found that monoclonal gammopathies are age‐related and affects men more than women, also the results of this study point to the delayed diagnosis of monoclonal gammopathies, since most of our patients were diagnosed at the MM stage. The most frequent isotypes were the IgGκ and IgGλ in MM and MGUS, in Waldenström macroglobulinemia were IgMκ and IgMλ and the oligoclonal profile represented only 3.70%.

## 1 INTRODUCTION

Monoclonal gammopathies (MG) represent a group of diseases characterized by a clonal proliferation of plasma cells that produce a monoclonal protein (M‐protein). The screening by using serum protein electrophoresis allowed their diagnosis at early stages. The prevalence of MG increases with age, several studies have reported a mean age at the time of diagnosis of 68 years, and most of them (99%) were over 40 years old.^1^, ^2^ It affects men more than women,^3^ and black race more than white. In a South African study on the prevalence of MGUS (Monoclonal gammopathy of undetermined signification) among 386 Black men, the prevalence of MGUS was significantly higher (8.03% [95% confidence interval (CI), 5.32–10.74]) in comparison to white men from Olmsted in the United States (2.97%, (95% CI, 2.59–3.34)).^4^

Monoclonal gammopathies of undetermined significance is defined as the production of monoclonal protein without any systemic effect. It represents the early stage of multiple myeloma (MM). MGUS accounts for 3% of MG in patients over 50 years of age.^5^ In general, MGUS will progress to MM at a rate of 1% per year.^6^ MM represents 10% of hematologic malignancies in general, the median age at diagnosis is estimated to be 70 years, and it affects more men (7 per 100 000) than women (4.5 per 100 000), blacks more than whites, thus, the highest prevalence rate is observed in African Americans, particularly in those aged 80–84 years and older.^2^ Some mutations and cytogenetic abnormalities are associated with high‐risk MM, such as translocations (*t*(4;14), *t*(14;16), *t*(14;20)), deletions: del (17p), gain 1q, or p53 mutation.^7^

The main objective was to describe the epidemiological and immunochemical characteristics of MG diagnosed over 19 years at a Moroccan teaching hospital.

## 2 MATERIALS AND METHODS

### 2.1 Patient selection

**FIGURE 1:**
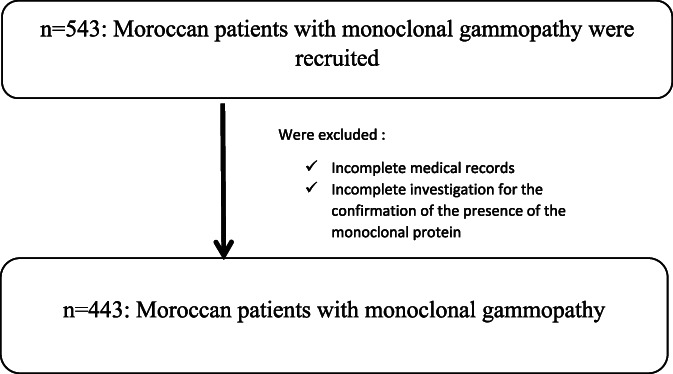
Flow chart which represents the study protocol.

The study was carried out over 19 years at the biochemistry department of Military Hospital in Rabat, the capital of Morocco, from January 2000 to August 2019. It was a retrospective study involving 443 Moroccan patients with monoclonal gammopathy detected by serum protein electrophoresis and serum/urine immunofixation. Patients included in the study have had a confirmed monoclonal protein at the serum electrophoresis with no missing serum/urine immunofixation and/or incomplete medical records. From 543 patients involved, only 443 patients were recruited. All participants signed an informed consent document (Figure 1).

### 2.2 Methods

Blood samples were collected using serum dry tubes, the samples were left to clot for 1 hour before centrifugation at 1000 RPM for 15 min. Total protein (g/L), C‐reactive protein (mg/L), Calcium (mg/L), creatinine (mg/L), and urea (g/L) were measured with Dimension RXL SIEMENS® analyzer. The β_2_‐microglobulin and immunoglobulins (IgA, IgG, and IgM) were measured with Immunonephelometer analyzers from DADE BEHRING BN II®. The Sedimentation rate was measured at the first hour (mm) (SRFH). Hemoglobin (Hb) (g/dL) was measured with the Beckman Coulter® hematology analyzer (reference values: In women Hb ˂12 g/dL, in men Hb˂13 g/dL). Serum protein electrophoresis were performed on gel and capillary electrophoresis SEBIA® to detect the presence of the monoclonal protein and to quantify it. To further characterize the patient's monoclonal protein, serum immunofixation electrophoresis and urine immunofixation electrophoresis were performed on SEBIA immunofixation analyzer®. For urine immunofixation electrophoresis only samples collected for 24 h was accepted.

Immunofixation was performed when an abnormal serum protein electrophoresis was defined by the presence of a quantifiable monoclonal spike, hypogammaglobulinemia, and a fuzzy band.

Patient records have been reviewed for clinical history and the monoclonal protein subtype. The diagnosis of MG is based on the presence of an increase in the number of plasma cells and/or immature and dystrophic plasma cells in the marrow, the presence of histological evidence of plasmacytoma, the presence of monoclonal protein in the serum and urine, and/or bone lesions.

### 2.3 Statistical analysis

The frequencies of clinical signs at admission and types of plasma cell dyscrasias were calculated using the software SPSS 13.0 of IBM for windows. The quantitative variables are presented as mean ± standard deviation (*SD*).

## 3 RESULTS

The baseline characteristics of the study participants (*n* = 443) are as follows. The study involved 320 (72.23%) men and 123 (27.77%) women, with mean ± *SD* age of 62.24 ± 13.14 years. The mean ± *SD* age by type of GM, MM, MGUS, WM, LLMM, KLMM, and POEMS (Polyneuropathy, organomegaly, endocrinopathy, monoclonal gammopathy, and skin changes syndrome) were respectively 61.70 ± 11.66 years, 63.64 ± 13.66 years, 61.71 ± 13.35 years, 60.57 ± 10.90 years, 56.50 ± 12.83 years and 45.50 ± 2.12 years. Regarding ethnic origin, all patients were of Caucasian origin, from 12 Moroccan regions. The most frequent reasons for admission were bone pain (41.60%), renal failure (19.08%), alteration of the general condition (12.21%), and anemia (10.69) (Table 1). The distribution of the MG was MM (45.65%), MGUS (39.05%), Waldenström macroglobulinemia (5.58%), Lymphoma (2.27% + 1.2%), Chronic Lymphocytic Leukemia (2.48%), Plasma cell leukemia (1.86%), Plasmacytoma (0.62%), POEMS syndrome (0.41%) and Amyloidosis (0.84%) (Table 2).

**TABLE 1:** Reasons for the acute medical admission.

	*n*	%
Bone pain	109	41.60
Renal failure	50	19.08
Anemia	28	10.69
Alteration of the general condition	32	12.21
Skin rash	1	0.38
Nephrotic syndrome	6	2.29
Tumor syndrome	8	3.05
Fracture	10	3.82
Hypercalcemia	6	2.29
Sedimentation rate at the first hour	6	2.29
Pancytopenia	6	2.29
	262	

**TABLE 2:** Repartition of the plasma cell proliferative disorders in this study.

	*n*	%
MM	221	45.65
MGUS	189	39.05
MW	27	5.58
PCL	9	1.86
PL	3	0.62
NHL	11	2.27
HL	6	1.24
CLL	12	2.48
POEMS	2	0.41
AL	4	0.84
	484	

Abbreviations: AL, amyloidosis; CLL, chronic lymphocytic leukemia; HL, Hodgkin lymphoma; MGUS, monoclonal gammopathies of undetermined signification; MM, multiple myeloma; MW, Waldenström's macroglobulinemia; NHL, non‐Hodgkin lymphoma; PCL, plasma cell leukemia; PL, plasmocytoma; POEMS, polyneuropathy, organomegaly, endocrinopathy, monoclonal protein and skin changes.

The estimated glomerular filtration rate (eGFR) in patients with MM was low (55.05 mL/min/1.73 m^2^) compared to other MG, but the lowest eGFR was in patients with Kappa Free light chain MM (19.33 mL/min/1.73 m^2^), more cases with kappa versus lambda light chain myeloma. The frequency of hypercalcemia was very high in patients with multiple myeloma (100.68 ± 18.79) and plasma cell leukemia (105.43 ± 11.10). The kappa/Lambda ratio was elevated in patients with MM (25.04), plasma cell leukemia (26.67) plasmacytoma (19.00), and MGUS.

The total protein level is highly increased in patients diagnosed with MM (38.21 g/L ± 23.83), MW (33.31 g/L ± 20.65), and MGUS (11.76 g/L ± 6.96), and low in patients diagnosed with light chain MM. Low albumin concentration was found in patients with MM and plasma cell leukemia.

Concentrations of parameters used in the prognostic evaluation of MG (β_2_‐microglobulin, CRP, and LDH) were higher in patients with MM and MGUS respectively. Anemia was more profound in patients with multiple myeloma in general (9.76 g/dL ± 2.21) and in patients with free light chains MM (LLMM [7.76 g/dL ± 1.12], KLMM [9.07 g/dL ± 1.81]) and Waldenström macroglobulinemia (9.29 g/dL ± 2.15) (Table 3).

**TABLE 3:** Characteristics of the patients with monoclonal protein in this study.

		Age (years)	eGFR mL/min/1.73 m^2^	Ca^2+^ (mg/L) (*N*: 90–105 mg/L)	Hb (g/dL)	(*N*: Men: 3 à 10 mm Women: 5 à 20 mm)	PU (g/24 h)	IgG (g/L) (*N*: 8 to 18 g/L)	IgA (g/L) (*N*: 0.9 to 4.50 g/L)	IgM (g/L) (*N*: 0.6 to 2.6 g/L)	Κ (<0.9 mg/dL)	Λ (<0.9 mg/dL)	κ/λ	MP (g/L)	TP (g/L) (*N*: 60 à 80 g/L)	ß2 microglobulin (mg/L)	LDH (135‐214UI/L)	CRP (mg/L) (*N*: 0–8 mg/L)	Albumin (g/L) (*N*: 35 to 55 g/L)
LLMM (*n* = 23)	*m* ± *SD*	60.57 ± 10.90	54.71 ± 51.86	104.30 ± 19.83	7.76 ± 1.12	50.07 ± 51.84	3.87 ± 4.26	6.28 ± 2.15	0.69 ± 0.74	0.37 ± 0.35	1.30 ± 0.43	4.59 ± 3.94	0.52 ± 0.51	7.50 ± 4.94	59.65 ± 13.91	18.97 ± 38.74	341.71 ± 216.46	67.01 ± 65.85	33.67 ± 5.51
	*n*	*n* = 23	*n* = 7	*n* = 23	*n* = 9	*n* = 14	*n* = 15	*n* = 10	*n* = 9	*n* = 7	*n* = 5	*n* = 5	*n* = 6	*n* = 8	*n* = 23	*n* = 11	*n* = 7	*n* = 18	*n* = 3
KLMM (*n* = 16)	*m* ± *SD*	56.50 ± 12.83	19.33 ± 23.97	102.00 ± 16.41	9.07 ± 1.81	69.91 ± 48.62	1.56 ± 1.31	5.64 ± 3.25	0.71 ± 0.53	0.46 ± 0.28	2.46 ± 1.51	1.56 ± 1.38	2.97 ± 2.14	0.00±	61.48 ± 10.39	8.14 ± 4.70	288.00 ± 102.83	47.79 ± 50.02	
	*n*	*n* = 16	*n* = 3	*n* = 15	*n* = 7	*n* = 11	*n* = 8	*n* = 8	*n* = 6	*n* = 4	*n* = 5	*n* = 4	*n* = 4	*n* = 1	*n* = 20	*n* = 7	*n* = 3	*n* = 10	
NSM (*n* = 5)	*m* ± *SD*	57.80 ± 11.21	22.00±	105.75 ± 27.84	7.70±	35.67 ± 38.42	3.26 ± 3.88	4.45 ± 0.31	0.67 ± 0.18	0.32 ± 0.03	0.91 ± 0.08	0.52 ± 0.01	1.88±		53.40 ± 1.14	1.65±	221.00±	18.95 ± 21.85	
	*n*	*n* = 5	*n* = 1	*n* = 4	*n* = 1	*n* = 3	*n* = 2	*n* = 3	*n* = 3	*n* = 3	*n* = 2	*n* = 2	*n* = 1		*n* = 5	*n* = 1	*n* = 1	*n* = 2	
MM (*n* = 164)	*m* ± *SD*	61.70 ± 11.66	55.05 ± 33.30	100.68 ± 18.79	9.76 ± 2.21	72.71 ± 41.26	2.13 ± 2.62	24.13 ± 22.89	12.00 ± 20.96	0.59 ± 0.97	7.77 ± 11.70	82.58 ± 57.87	25.04 ± 46.20	38.21 ± 23.83	87.33 ± 23.31	19.23 ± 44.30	259.43 ± 176.48	43.74 ± 71.85	26.39 ± 5.97
	*n*	*n* = 164	*n* = 55	*n* = 132	*n* = 91	*n* = 100	*n* = 65	*n* = 71	*n* = 76	*n* = 69	*n* = 59	*n* = 53	*n* = 54	*n* = 153	*n* = 164	*n* = 74	*n* = 42	*n* = 117	*n* = 23
PCL (*n* = 8)	*m* ± *SD*	57.75 ± 10.99	67.50 ± 21.24	105.43 ± 11.10	8.16 ± 3.58	53.50 ± 48.43	1.30 ± 0.83	20.61 ± 30.68	0.22 ± 0.04	0.21 ± 0.03	6.04 ± 9.24	1.91 ± 1.48	26.67 ± 45.76	30.90 ± 16.21	69.63 ± 18.64	14.44 ± 5.24	209.25 ± 67.23	66.60 ± 63.35	24.00 ± 4.24
	*n*	*n* = 8	*n* = 4	*n* = 7	*n* = 5	*n* = 4	*n* = 3	*n* = 3	*n* = 3	*n* = 3	*n* = 3	*n* = 3	*n* = 3	*n* = 6	*n* = 11	*n* = 5	*n* = 4	*n* = 3	*n* = 2
MGUS (*n* = 172)	*m* ± *SD*	63.64 ± 13.66	63.46 ± 29.09	91.70 ± 8.79	11.74 ± 2.55	39.28 ± 25.86	0.86 ± 1.57	14.62 ± 7.81	4.12 ± 4.72	1.08 ± 1.11	3.37 ± 1.77	2.20 ± 1.66	2.06 ± 1.50	11.76 ± 6.96	70.06 ± 11.58	6.20 ± 6.66	211.56 ± 80.21	43.89 ± 63.74	30.50 ± 7.22
	*n*	*n* = 172	*n* = 80	*n* = 141	*n* = 100	*n* = 84	*n* = 58	*n* = 57	*n* = 53	*n* = 55	*n* = 45	*n* = 43	*n* = 41	*n* = 148	*n* = 6	*n* = 33	*n* = 50	*n* = 125	*n* = 20
MW(*n* = 21)	*m* ± *SD*	61.71 ± 13.35	76.54 ± 35.35	98.20 ± 10.26	9.29 ± 2.15	107.80 ± 32.39	0.45 ± 0.40	11.70 ± 8.06	3.84 ± 6.25	35.30 ± 31.67	3.98 ± 2.46	2.89 ± 2.67	2.89 ± 1.84	33.31 ± 20.65	79.40 ± 21.67	5.05 ± 2.39	314.50 ± 167.08	68.36 ± 71.90	27.00 ± 4.20
	*n*	*n* = 21	*n* = 13	*n* = 15	*n* = 18	*n* = 10	*n* = 7	*n* = 13	*n* = 14	*n* = 11	*n* = 7	*n* = 7	*n* = 5	*n* = 20	*n* = 27	*n* = 8	*n* = 12	*n* = 16	*n* = 6
NHL(*n* = 10)	*m* ± *SD*	59.50 ± 15.15	68.25 ± 19.02	90.40 ± 12.58	11.37 ± 2.32	42.20 ± 31.31	0.67 ± 0.33							14.25 ± 1.97	62.44 ± 12.00	5.99 ± 3.92	287.50 ± 88.95	69.35 ± 91.40	25.00±
	*n*	10	4	5	6	5	2							6	12	3	4	6	1
HL (*n* = 5)	*m* ± *SD*	50.20 ± 24.19	43.00±	92.75 ± 2.75	9.30 ± 2.69	20.00 ± 14.14								13.66 ± 6.65	73.66 ± 8.32	2.61 ± 1.82	237.00±	59.23 ± 98.19	
	*n*	*n* = 5	*n* = 1	*n* = 4	*n* = 2	*n* = 2								*n* = 5	*n* = 3	*n* = 2	*n* = 1	*n* = 3	
CLL (*n* = 10)	*m* ± *SD*	63.60 ± 18.50	93.20 ± 14.13	92.25 ± 7.52	9.48 ± 1.73	14.00 ± 5.66								7.45 ± 6.21	77.88 ± 22.46	5.63 ± 3.92	1088.40 ± 1182.08	57.26 ± 55.26	28.00±
	*n*	*n* = 10	*n* = 5	*n* = 8	*n* = 8	*n* = 2								*n* = 6	*n* = 9	*n* = 2	*n* = 5	*n* = 7	*n* = 1
PL (*n* = 3)	*m* ± *SD*	66.00 ± 15.52	89.00±	89.67 ± 4.62	15.40±	67.00 ± 42.15	0.07±	15.22 ± 18.08	19.00±	0.17±	7.98±	0.42±	19.00±	18.22 ± 10.00	68.75 ± 9.07		182.00±	52.65 ± 70.92	
	*n*	*n* = 3	*n* = 1	*n* = 3	*n* = 1	*n* = 3	*n* = 1	*n* = 2	*n* = 1	*n* = 1	*n* = 1	*n* = 1	*n* = 1	*n* = 3	*n* = 186		*n* = 1	*n* = 2	
POEMS (*n* = 2)	*m* ± *SD*	45.50 ± 2.12						7.65 ± 4.41	8.70 ± 11.74	0.99 ± 1.17	1.31 ± 0.60	2.10 ± 1.43	0.69 ± 0.18	21.20±	60 ± 19.79				
	*n*	*n* = 2						*n* = 2	*n* = 2	*n* = 2	*n* = 2	*n* = 2	*n* = 2	*n* = 1	*n* = 2				
AL (*n* = 4)	*m* ± *SD*	54.25 ± 7.41	51.50 ± 47.37	78.66 ± 24.54	10.25 ± 3.00	60.00±	6152 ± 5.52	8.40 ± 0.87	3.86 ± 1.25	0.7250 ± 0.65	2.76 ± 0.12	1.81 ± 0.56	1.59 ± 0.43	11.00±	54.33 ± 13.05	19.44 ± 1.75	317.00 ± 5.65	34.12 ± 42.04	
	*n*	*n* = 4	*n* = 2	*n* = 3	*n* = 4	*n* = 1	*n* = 4	*n* = 2	*n* = 2	*n* = 2	*n* = 2	*n* = 2	*n* = 2	*n* = 1	*n* = 3	*n* = 2	*n* = 2	*n* = 4	

*Note*: *m ± SD*.

Abbreviations: AL, amyloidosis; CLL, chronic lymphocytic leukemia; CRP, C‐reactive protein; eGFR, estimated glomerular filtration rate (modification of diet in renal disease [MDRD]); HL, Hodgkin lymphoma; KLMM, kappa light chain multiple myeloma; LLMM, lambda light chain multiple myeloma; MGUS, monoclonal gammopathies of undetermined signification; MM, multiple myeloma; MW, Waldenström's macroglobulinemia; NHL, non‐Hodgkin lymphoma; NSM, non‐secretant multiple myeloma; PCL, plasma cell leukemia; PL, plasmocytoma; POEMS, polyneuropathy, organomegaly, endocrinopathy, monoclonal protein and skin changes; PU, proteinuria; SRFH, sedimentation rate at the first hour (mm); TP, total protein.

The isotype repartition by Plasma cell proliferative disorders in this study is given in Table 4. The most frequent isotype in MM were the IgGκ (62) 36.5%, IgGλ (52) 30.6%, IgAκ (27) 15.9% and the IgAλ (19) 11.2%. It is also worth noting that Free light chain MM represents 20% of all cases of MM.

**TABLE 4:** The number of different types of paraprotein detected in patients versus monoclonal gammopathies diagnostics.

	MM	MGUS	PCL	PL	LLMM	KLMM	MW	NHL	HL	CLL	POEMS	AL	NSM
IgGκ (*n*)%	(62) 36.5	(87) 46.27	(2) 25	(0) 0	(0) 0	(0) 0	(0) 0	(2) 22.22	(0) 0	(4) 33.3	(0) 0	(0) 0	(0) 0
IgGλ (*n*)%	(52) 30.6	(61) 32.44	(3) 37.5	(2) 75	(0) 0	(0) 0	(0) 0	(2) 22.22	(1) 17	(0) 0	(0) 0	(2) 67	(0) 0
IgAκ (*n*)%	(27) 15.9	(4) 2.13	(0) 0	(1) 25	(0) 0	(0) 0	(0) 0	(0) 0	(0) 0	(0) 0	(0) 0	(0) 0	(0) 0
IgAλ (*n*)%	(19) 11.2	(17) 9.04	(1) 12.5	(0) 0	(0) 0	(0) 0	(0) 0	(0) 0	(0) 0	(1) 8.33	(1) 50	(0) 0	(0) 0
IgMK (*n*)%	(0) 0	(9) 4.78	(0) 0	(0) 0	(0) 0	(0) 0	(19) 70.37	(3) 33.33	(2) 33	(4) 33.3	(0) 0	(0) 0	(0) 0
IgMλ (*n*)%	(0) 0	(5) 2.66	(0) 0	(0) 0	(0) 0	(0) 0	(7) 25.93	(1) 11.11	(1) 17	(0) 0	(0) 0	(0) 0	(0) 0
IgDκ (*n*)%	(1) 0.59	(0) 0	(0) 0	(0) 0	(0) 0	(0) 0	(0) 0	(0) 0	(0) 0	(0) 0	(0) 0	(0) 0	(0) 0
IgDλ (*n*)%	(2) 1.18	(0) 0	(0) 0	(0) 0	(0) 0	(0) 0	(0) 0	(0) 0	(0) 0	(0) 0	(0) 0	(0) 0	(0) 0
CLLK (*n*)%	(0) 0	(0) 0	(0) 0	(0) 0	(0) 0	(19) (100)	(0) 0	(0) 0	(0) 0	(0) 0	(1) 50	(0) 0	(0) 0
CLLL (*n*)%	(0) 0	(0) 0	(2) 25	(0) 0	(22) 100	(0) 0	(0) 0	(0) 0	(0) 0	(1) 8.33	(0) 0	(0) 0	(0) 0
IgGκ + IgAλ (*n*)%	(1) 0.59	(1) 0.53	(0) 0	(0) 0	(0) 0	(0) 0	(0) 0	(0) 0	(0) 0	(1) 8.33	(0) 0	(0) 0	(0) 0
IgMκ + IgGκ (*n*)%	(0) 0	(0) 0	(0) 0	(0) 0	(0) 0	(0) 0	(0) 0	(0) 0	(1) 17	(0) 0	(0) 0	(0) 0	(0) 0
IgAλ + IgMκ (*n*)%	(0) 0	(1) 0.53	(0) 0	(0) 0	(0) 0	(0) 0	(0) 0	(0) 0	(0) 0	(0) 0	(0) 0	(0) 0	(0) 0
IgGκ + IgGλ (*n*)%	(0) 0	(1) 0.53	(0) 0	(0) 0	(0) 0	(0) 0	(0) 0	(0) 0	(1) 17	(0) 0	(0) 0	(0) 0	(0) 0
IgGκ + IgAλ (*n*)%	(0) 0	(2) 1.06	(0) 0	(0) 0	(0) 0	(0) 0	(0) 0	(0) 0	(0) 0	(0) 0	(0) 0	(0) 0	(0) 0
Oligoclonal (*n*)%	(0) 0	(0) 0	(0) 0	(0) 0	(0) 0	(0) 0	(1) 3.70	(1) 11.11	(0) 0	(1) 8.33	(0) 0	(1) 33	(0) 0
Total	164	188	8	3	22	19	27	9	6	12	2	3	0

Abbreviations: AL, amyloidosis; CLL, chronic lymphocytic leukemia; HL, Hodgkin lymphoma; KLMM, kappa light chain multiple myeloma; LLMM, lambda light chain multiple myeloma; MGUS, monoclonal gammopathies of undetermined signification; MM, multiple myeloma; MW, Waldenström's macroglobulinemia; NHL, non‐Hodgkin lymphoma; NSM, non‐secretant multiple myeloma; PCL, plasma cell leukemia; PL, plasmocytoma; POEMS, polyneuropathy, organomegaly, endocrinopathy, monoclonal protein and skin changes.

For the MGUS, the most frequent isotypes were the IgGκ (87) 46.27%, IgGλ (61) 32.44%, IgAκ (4) 2.13%, IGAλ (9) 4.78%, IgMλ (5) 2.66% and IgMκ (9) 4.78%. Finally, the isotypes distribution in Waldenström macroglobulinemia were IgMκ (19) 70.37%, IgMλ (7) 25.93%, and an oligoclonal profile (1) 3.70% (Table 4).

## 4 DISCUSSION

The study was conducted over a period of 19 years between January 2000 and August 2019. The patients who were included were 543 patients. The main objective was to describe the epidemiological and immunochemical characteristics of MG diagnosed in a Moroccan teaching hospital for 19 years. The results of this study point to the need for an early diagnosis in the Moroccan population. The mean ± *SD* age at diagnosis of all MG was 62.24 years ±13.14 with a sex ratio male/female of 2.14. Our results are in agreement with the results of international studies confirming that MG is age‐related and affects men more than women.^8^, ^9^, ^10^

Plasma cell proliferative disorders in our study were as follows, MM 45.65% (*n* = 221), MGUS 39.05% (*n* = 189), WM 5.58% (*n* = 27), plasmacytomas 5.58% (*n* = 27), plasma cell leukemia 0.62% (*n* = 3). The most frequent diagnosis of MG in European and American studies was MGUS.^11^, ^12^ In contrast, MM was the most frequent monoclonal gammopathy in this study and the studies from Algeria and Tunisia.^13^, ^14^ This is due to the delay in diagnosis and the lack of serum protein electrophoresis in most of the hospitals in Maghreb countries. In a cross‐sectional study, Gupta et al reported a low incidence of MGUS in Indians compared to black and white populations. This study included 3429 patients, and only 49 (1.43%) had a diagnosis of MGUS at the time of diagnosis.^15^ The risk factors for the development of MGUS are the first‐degree relatives of patients with MM, obesity, the population older than 50 years, ethnicity, and the exposition to pesticides, radiation, and petroleum products.^16^

In this study, the POEMS syndrome was diagnosed only in 2 patients (0.41%), POEMS syndrome is relatively rare, in a mayo clinic study only 99 cases were diagnosed.^17^ The pathophysiology of the neuropathy in the POEMS syndrome was associated with the alteration in sodium and potassium channels present in the nodes of Ranvier and axon and the imbalance of increased VEGF (Vascular Endothelial Growth Factors) and decreased serum erythropoietin.^18^

The κ/λ ratio was very high in patients with MM (25.04), plasma cell leukemia (26.67), MGUS (2.06), and plasmacytoma (19.00). Indeed, in patients with MM, Belouni et al, Mseddi et al and the Mayo Clinic^12^, ^13^, ^14^ have found the kappa/Lambda ratio respectively of 14.9, 14.13, and 16. Nevertheless, the normal κ/λ ratio does not exclude the presence of a monoclonal gammopathy according to Singh et al, and false negatives rates associated with lambda chains are higher than those for lesions with kappa chains.^19^

The mean concentrations of the monoclonal peak were MM (38.21 ± 23.83), MW (33.31 ± 20.65), and MGUS (11.76 ± 6.96), these results are very far from the means noted in the patients included in the Algerian study of Belouni et al (22.19 ± 20.19 g/L)^13^ and the majority of the other series, this can be explained by the late diagnosis of the MG in our country, and the high frequency of MM in our study in comparison of the international studies.^11^, ^12^ Furthermore, Monoclonal protein concentration was elevated in patients diagnosed with MW or MM compared to MGUS.

Anemia was more profound in patients with free light chain MM, LLMM (7.76 ± 1.12), and KLMM (9.07 ± 1.81). This may be explained by the high frequency of free light chain MM, and the delay in the diagnosis of our patients with MM.

The isotype repartition by plasma cell proliferative disorders is given in Table 4. The most frequent monoclonal proteins in the group of patients with MM were the IgGκ (62) 36.5%, IgGλ (52) 30.6%, IgAκ (27) 15.9% and the IgAλ (19) 11.2%. Regarding the MGUS group, the most frequent isotypes were the IgGκ (87) 46.27%, IgGλ (61) 32.44%, IgAκ (4) 2.13%, IgAλ (9) 4.78%, IgMλ (5) 2.66% and IgMκ (9) 4.78%. The same results were reported in the study of Landgren et al, the most frequent isotypes by race/ethnicity were IgG %(*n*) 76.1 (71), IgA 5.2 (5), IgM 2.7 (3) in black, IgG 68.1 (150), IgA 9.7 (12), IgM 15.4 (34) in whites and IgG 63.1 (29) IgA 16.7(8) IgM 7.4 (6) in Mexican Americans.^20^ Finally, the most frequent isotypes in Waldenström macroglobulinemia were IgMκ (19) at 70.37%, and IgMλ (7) at 25.93%. These results are consistent with the results of studies from the Maghreb countries, in which the most common isotypes were the IgG followed by the Ig A and the IgM in the studies of Mseddi et al in Tunisia, and Belouni et al in Algeria.^13^, ^14^

Our study had strengths and weaknesses, the strengths were represented by a large number of patients with MG, and the length of follow‐up of these patients. The limitations were represented by the retrospective type of the study.

## 5 CONCLUSION

This is the largest Moroccan cohort, it included 443 patients. Our results are in agreement with the results of international studies confirming that GM is age‐related and affects men more than women. The most frequent plasma cell proliferative disorder at the diagnosis was MM 45.65% (*n* = 221), this is due to the delay in diagnosis. Therefore, we recommend that Moroccan doctors prescribe a serum protein electrophoresis for people over 50 years of age and especially for people with professional exposure to toxic substances (oil, pesticides…). The results of this study point to the need for an early diagnosis of MG in the Moroccan population. The most frequent isotypes were the IgGκ and IgGλ in MM and MGUS, in Waldenström macroglobulinemia were IgMκ and IgMλ and the oligoclonal profile represented only 3.70%. It is also worth noting that Free light chain MM represents 20% of all cases of MM.

## AUTHOR CONTRIBUTIONS

All the authors have seen and approved the final manuscript. All authors meet the criteria of authorship. Extracted the clinical and lab data: Kamal Doghmi, Nezha Messaoudi, Sanae Bouhsain, Samira El Machtani, Ahmed Bezza, Achraf Rachid, Abdallah Dami, Asmae Biaz, Aissam El Maataoui; Acquisition of data: Aissam El Maataoui; Analysis and interpretation of data: Aissam El Maataoui; Drafting of the manuscript: Aissam El Maataoui; Critical revision: Zohra Ouzzif, Aissam El Maataoui.

## CONFLICT OF INTEREST STATEMENT

The authors have stated explicitly that there are no conflicts of interest in connection with this article.

## ETHICS STATEMENT

The local ethics committee at Mohamed V University, Faculty of Medicine and Pharmacy Rabat approved this study. Patients were recruited and enrolled at Mohamed V Teaching Hospital in Rabat. Written informed consent procedures, conforms to the ethical guidelines of the Declaration of Helsinki and were approved by the ethics committee at Mohamed V University, Faculty of Medicine and Pharmacy, Rabat‐Morocco.

## Data Availability

The data that support the findings of this study are availabe from the corresponding author upon reasonable request.
